# Undiagnosed placenta accreta spectrum complicated by massive haemorrhage during mid-trimester medical termination of pregnancy: a case report

**DOI:** 10.1016/j.crwh.2026.e00790

**Published:** 2026-02-17

**Authors:** Natalie Drever, Sunthra Shanmuga Lingam, Joanna Arnold

**Affiliations:** a James Cook University, Cairns, Queensland, Australia; b Cairns Hospital, Queensland, Australia

**Keywords:** Placenta accreta, Abortion, Obstetric haemorrhage, Emergency hysterectomy, Maternal morbidity

## Abstract

Placenta accreta spectrum (PAS) is a well-recognised cause of severe obstetric haemorrhage in late pregnancy but is rarely encountered during abortion, particularly in the mid-trimester. Diagnosis in this setting is challenging, and unrecognised abnormal placentation may result in catastrophic maternal morbidity. We report the case of a 28-year-old multiparous woman with three prior caesareans who presented at 17 weeks of gestation with preterm prelabour rupture of membranes and placenta previa. Targeted mid-trimester ultrasound reviewed by a maternal–fetal medicine subspecialist demonstrated no sonographic features suggestive of PAS. Following counselling, medical termination of pregnancy was initiated using mifepristone and misoprostol. Uncontrolled bleeding led to timely transfer to the operating theatre for hysterotomy and management of haemorrhage. The procedure was complicated by sudden massive haemorrhage, haemodynamic collapse, and intraoperative cardiac arrest. Despite uterine evacuation and resuscitative measures, uncontrolled bleeding necessitated an emergency subtotal hysterectomy, complicated by dense vesicouterine adhesions and bladder injury. The patient survived following massive transfusion, intensive care admission, and multidisciplinary management. This case highlights the limitations of mid-trimester imaging in reliably excluding clinically significant PAS. It also underscores the potential for abrupt, life-threatening haemorrhage during medical termination of pregnancy in women with multiple previous caesarean deliveries. As caesarean rates rise, clearer guidance is needed regarding risk stratification, imaging pathways, and procedural planning for mid-trimester abortion to minimise maternal morbidity.

## Highlights

• Placenta accreta encountered during abortion may lead to severe haemorrhage.
• Mid-trimester diagnosis of accreta remains challenging despite targeted ultrasound.
• Placenta praevia with prior caesarean should raise suspicion of accreta.
• Outcomes appear worse when placenta accreta is unrecognised prior to abortion.
• Limited evidence guides abortion method after multiple prior caesareans.

## 1 Introduction

Placenta accreta spectrum (PAS) is a well-recognised cause of severe obstetric haemorrhage and maternal morbidity, typically identified on imaging in late pregnancy and managed with planned delivery in the third trimester [1], [2]. In contrast, PAS complicating abortion remains poorly characterised, particularly in the mid-trimester [3]. When PAS is unrecognised prior to abortion, haemorrhage may be sudden, severe, and life-threatening [3], [4].

Reports of PAS encountered during abortion are rare and predominantly involve first-trimester caesarean scar pregnancy (CSP) or surgical termination. A recent scoping review identified only a small number of case reports describing undiagnosed PAS encountered during abortion. Emergency hysterectomy was required in 8 of 11 published cases; however, most involved first-trimester CSP or surgical abortion rather than mid-trimester medical termination of pregnancy (MToP) [3]. Only a small number of case reports describe mid-trimester termination complicated by concurrent accreta; in one of these, by Matsuzaki et al., suspected accreta was associated with massive haemorrhage requiring hysterectomy [5].

Importantly, evidence suggests that outcomes differ substantially depending on whether PAS is recognised prior to abortion. When abnormal placentation is diagnosed antenatally, abortion may be planned with multidisciplinary input, with or without interventional radiology [6], [7], [8].

Current clinical guidelines acknowledge the increased risk of PAS in women undergoing second-trimester abortion after prior caesarean and recommend targeted mid-trimester ultrasound assessment [9]. However, there is limited guidance regarding optimal imaging modality, the role of magnetic resonance imaging (MRI), or the required level of expertise of the operator. Similarly, guidance on the preferred method of abortion in women with multiple prior caesareans remains limited. Surgical abortion may become technically more complex with multiple prior caesareans, while MToP carries risks, including uterine rupture, which appear to increase with gestational age and increasing number of prior caesareans [3].

As caesarean rates continue to rise [10], increasing numbers of women with multiple uterine scars will present for mid-trimester abortion. This case highlights the potential for catastrophic haemorrhage due to undiagnosed PAS during MToP. It underscores the limitations of current diagnostic pathways and the need for clearer guidance regarding imaging, risk stratification, and procedural planning for mid-trimester abortion in women with multiple prior caesareans.

## 2 Case Presentation

A 28-year-old woman (gravida 14, para 3) presented to a large regional hospital at 17 weeks of gestation with preterm prelabour rupture of membranes (PPROM). Her obstetric history was notable for three prior term lower-segment caesareans, the first an emergency caesarean for obstructed labour, followed by two uncomplicated repeat elective caesareans. She had experienced recurrent early pregnancy loss (one requiring dilation and curettage), and a previous second-trimester MToP complicated by retained placenta requiring manual removal without haemorrhage. She had no medical comorbidities and no non-obstetric surgical history. This was a planned pregnancy conceived spontaneously. She received obstetric and midwifery care through the public hospital and had an otherwise uncomplicated antenatal course. Prior ultrasound imaging was a single first-trimester dating ultrasound that had not demonstrated features suspicious for abnormal placentation.

She initially presented with several days of myalgia, malaise, and clear per-vaginal fluid loss without purulent discharge. On admission, she was febrile though otherwise haemodynamically stable. Abdominal examination revealed a soft, non-tender abdomen. Speculum examination demonstrated blood-stained fluid in the vagina, with the cervix 0.5 cm dilated and no purulent discharge.

Bedside ultrasound confirmed anhydramnios with a live intrauterine pregnancy. Investigations showed leukocytosis and an elevated C-reactive protein. Formal ultrasound demonstrated a live fetus consistent with 17 weeks of gestation, cephalic presentation, a cervical length of approximately 2.8 cm and a complete placenta previa. The images were reviewed by a maternal–fetal medicine subspecialist, and no sonographic markers of PAS were identified. Based on imaging criteria, the risk of placenta accreta spectrum was assessed as low.

The diagnosis of previable PPROM was discussed with the patient, including the poor fetal prognosis and the maternal risks associated with expectant management, particularly if clinical chorioamnionitis developed. At the time of counselling, there was no definitive clinical evidence of intrauterine infection, though the potential for clinical deterioration was acknowledged. She was admitted and commenced on broad-spectrum intravenous antibiotics.

Following further counselling, the patient elected to proceed with termination of pregnancy the following day. MToP was initiated with 200 mg oral mifepristone, followed by inpatient misoprostol induction 36 h later. Three hours after the first dose of 400μg sublingual misoprostol, the patient developed sudden heavy vaginal bleeding associated with hypotension. Initial estimated blood loss exceeded 1000 mL, and she required urgent fluid resuscitation and transfer to theatre for examination under anesthesia and further surgical management.

Due to ongoing haemorrhage, a hysterotomy was performed to facilitate delivery of the fetus and placenta. Despite uterine evacuation and attempted mechanical tamponade, massive haemorrhage persisted. During the procedure, the patient developed haemodynamic collapse and sustained a cardiac arrest requiring cardiopulmonary resuscitation. Return of spontaneous circulation was achieved following advanced life support measures and massive transfusion. Uncontrolled haemorrhage necessitated an emergency subtotal hysterectomy as a life-saving intervention.

The hysterectomy was complicated by dense vesicouterine adhesions, resulting in inadvertent bladder injury, which was repaired intraoperatively with urological assistance and ureteric stenting. Total estimated blood loss was 4500 mL, and the patient required 12 units of packed red blood cells and additional blood products. Following stabilisation, she was transferred to the intensive care unit.

Postoperatively, the patient made a full recovery. She was counselled regarding the diagnosis of suspected PAS, intraoperative cardiac arrest, and the necessity for emergency hysterectomy to control life-threatening haemorrhage. She expressed a desire to see her baby, and bereavement support was provided. She was discharged following recovery with planned gynaecological and urological follow-up. Staff involved in the patient's care were subsequently offered formal debriefing due to the psychologically distressing nature of the event. Histopathological examination of the hysterectomy specimen confirmed PAS.

## 3 Discussion

This case illustrates that PAS may remain undetected in the mid-trimester despite specialist ultrasound review and can present with abrupt, life-threatening haemorrhage during medical termination of pregnancy.

Only a small number of case reports describe mid-trimester termination complicated by concurrent placenta previa and PAS. Matsuzaki et al. reported a similar case of second-trimester termination following PPROM, in which placenta accreta was suspected on ultrasound and MToP using gemeprost was complicated by massive haemorrhage requiring hysterectomy [5]. In contrast, no sonographic features of accreta were identified in the present case despite maternal–fetal medicine specialist review, and MToP was undertaken using a contemporary mifepristone–misoprostol regimen. Together, these cases highlight that severe haemorrhage may occur both when PAS is suspected and when it remains unrecognised, and across different medical abortion protocols.

The diagnostic performance of mid-trimester imaging for PAS remains uncertain. While targeted ultrasound assessment is recommended for women with risk factors such as placenta previa and prior caesarean [2], the sensitivity of ultrasound earlier in gestation is lower than in late pregnancy [1], [11]. Guidance regarding the role of adjunct MRI [1], [12], optimal timing of assessment, and required operator expertise in the context of mid-trimester abortion is limited [9]. As illustrated by this case, absence of typical imaging features does not reliably exclude clinically significant abnormal placentation.

The optimal method of termination of pregnancy in women with multiple prior caesarean deliveries remains unclear, particularly in the mid-trimester. Evidence comparing medical and surgical approaches in this group is limited, and guidance on risk stratification is sparse [3]. While surgical evacuation may avoid uterine incision and, in theory, reduce bleeding compared with hysterotomy, feasibility is highly dependent on gestational age, cervical access, and the availability of appropriately trained providers. MToP, conversely, carries risks, including haemorrhage and uterine rupture, which appear to increase with advancing gestation [3]. Data to guide selection of termination method in women with three or more prior caesareans are lacking.

Reports describing management of PAS encountered during abortion include a range of strategies, such as planned hysterotomy, prophylactic interventional radiology, and hysterectomy, with variable outcomes. Kaba et al. recently highlighted the technical complexity of emergency hysterectomy in a woman with multiple prior caesarean deliveries, emphasising dense vesicouterine adhesions and the challenges of haemorrhage control [13]. Modified one-step conservative uterine surgery has also been described as a fertility-preserving approach in selected cases of antenatally diagnosed PAS [14]. However, this technique requires preoperative suspicion and haemodynamic stability. In the present case, PAS was not suspected on imaging, and sudden massive haemorrhage with cardiac arrest necessitated immediate life-saving hysterectomy. In regional settings where interventional radiology access may be limited, proactive planning becomes particularly important.

Early gravid hysterectomy may represent an appropriate option for selected women with suspected PAS who have completed their families and are undergoing abortion, potentially reducing the risk of uncontrolled haemorrhage [5], [13]. While this approach would not have been appropriate in the present case given the patient's age and desire for future fertility, it remains an important consideration in counselling and planning for other patients.

## 4 Conclusion

This case highlights that PAS may remain undetected in the mid-trimester despite specialist ultrasound assessment and can present with sudden, uncontrollable haemorrhage during medical termination of pregnancy. Outcomes appear more favourable when PAS is recognised and management is planned. As caesarean rates continue to rise, clearer guidance is required regarding imaging pathways, thresholds for tertiary referral, and selection of abortion method to minimise morbidity.

## Contributors

Natalie Drever contributed to conception of the case report, acquiring and interpreting the data, drafting the manuscript, undertaking the literature review and revising the article critically for important intellectual content.

Sunthra Shanmuga Lingam and Joanna Arnold contributed to patient care, interpreting the data and revising the article critically for important intellectual content.

All authors approved the final submitted version.

## Patient consent

Written informed consent was obtained from the patient for publication of this case report and associated clinical information. The patient was advised that identifying details would be removed and that anonymity would be maintained as far as possible.

## Provenance and peer review

This article was not commissioned and was peer reviewed.

## Declaration of generative AI and AI-assisted technologies in the writing process

During the preparation of this work, the authors used ChatGPT to assist with language refinement and structural editing. After using this tool, the authors reviewed and edited the content as needed and take full responsibility for the content of the publication.

## Funding

The publication of this case report did not receive any specific grant from funding agencies in the public, commercial, or not-for-profit sectors.

## Declaration of competing interest

The authors declare that they have no competing interest regarding the publication of this case report.
